# Expression of pIgR in the tracheal mucosa of SHIV/SIV-infected rhesus macaques

**DOI:** 10.13918/j.issn.2095-8137.2017.007

**Published:** 2017-01-18

**Authors:** Dong Li, Feng-Jie Wang, Lei Yu, Wen-Rong Yao, Yan-Fang Cui, Gui-Bo Yang

**Affiliations:** National Institute of AIDS/STD Control and Prevention, China-CDC, Beijing 102206, China; National Institute of AIDS/STD Control and Prevention, China-CDC, Beijing 102206, China; National Institute of AIDS/STD Control and Prevention, China-CDC, Beijing 102206, China; National Institute of AIDS/STD Control and Prevention, China-CDC, Beijing 102206, China; National Institute of AIDS/STD Control and Prevention, China-CDC, Beijing 102206, China; National Institute of AIDS/STD Control and Prevention, China-CDC, Beijing 102206, China

**Keywords:** Tracheal mucosa, Lungs, pIgR, SHIV/SIV infection, IL-17A

## Abstract

Polymeric immunoglobulin receptors (pIgR) are key participants in the formation and secretion of secretory IgA (S-IgA), which is critical for the prevention of microbial infection and colonization in the respiratory system. Although increased respiratory colonization and infections are common in HIV/AIDS, little is known about the expression of pIgR in the airway mucosa of these patients. To address this, the expression levels of pIgR in the tracheal mucosa and lungs of SHIV/SIV-infected rhesus macaques were examined by real-time RTPCR and confocal microscopy. We found that the levels of both *PIGR* mRNA and pIgR immunoreactivity were lower in the tracheal mucosa of SHIV/SIV-infected rhesus macaques than that in non-infected rhesus macaques, and the difference in pIgR immunoreactivity was statistically significant. IL-17A, which enhances pIgR expression, was also changed in the same direction as that of pIgR. In contrast to changes in the tracheal mucosa, pIgR and IL-17A levels were higher in the lungs of infected rhesus macaques. These results indicated abnormal pIgR expression in SHIV/SIV, and by extension HIV infections, which might partially result from IL-17A alterations and might contribute to the increased microbial colonization and infection related to pulmonary complications in HIV/AIDS.

## INTRODUCTION

The respiratory system is continuously exposed to foreign antigens from either airborne or commensal microbes. Due to vulnerability of the physical epithelial barrier of the respiratory system, most pathogens are stopped from entering the body by the mucosal immune system. A key component of the airway mucosal immune system that prevents microbial infections and colonization is secretory IgA (S-IgA), which is composed of dimeric IgA produced in the lamina propria and extracellular part of the polymeric immunoglobulin receptors (pIgR), also known as the secretory component (SC) expressed by mucosal epithelial cells (Johansen & Kaetzel, 2011). 

Newly synthesized pIgR is localized to the basolateral surfaces of mucosal epithelial cells, where it binds to dimeric IgA (dIgA) and mediates transcytosis of IgA to the apical surface of the epithelial cells (Johansen et al., 1999). The SC can be released to the mucosal surface alone (in the absence of IgA) or together with dIgA as S-IgA. In addition, SC bound to dIgA can elongate the life of S-IgA and enhance its immune exclusion ability. It can also stop microbial invasion. Mice deficient in pIgR expression are reportedly unable to control infections of the airway by some bacteria, which could drive progressive chronic obstructive pulmonary disease (COPD) phenotype in these mice (Richmond et al., 2016). 

Pulmonary complications are common and major causes of morbidity and mortality in HIV-infected individuals, even in the presence of highly active antiretroviral therapy (ART) (Grubb et al., 2006; Murray, 1996). Increased pulmonary infections and microbial colonization are common in HIV/AIDS patients (Zar, 2008). Whether and how the S-IgA/pIgR system is involved in these alterations is not well addressed. Rhesus macaques are important in HIV/AIDS studies. In previous research, we found that pIgR expression was altered in the gut mucosa of SHIV/SIV-infected rhesus macaques (Wang & Yang, 2016). To determine whether pIgR is involved in the respiratory pathology of HIV/AIDS, we examined the expression of pIgR in the tracheal mucosa of SHIV/SIV-infected rhesus macaques.

## MATERIALS AND METHODS

### Tissues

Tissue samples from the tracheas and lungs were collected from five normal and five SHIV/SIV-infected rhesus macaques (*Macaca mulatta*), as reported previously (Wang & Yang, 2016). The sites from which samples were collected were chosen randomly. Tissue samples for RNA isolation were frozen on dry ice immediately after collection and preserved in a freezer at -80 ℃ before use. Tissue samples for confocal microscopy were fixed in 4% paraformaldehyde immediately after collection, then washed and protected with 30% sucrose, and finally embedded in OCT and preserved in a freezer at -80 ℃. All study animals were treated humanely per the state and local regulations on the care and use of experimental animals.

### Real-time RT-PCR

Quantification of pIgR and IL-17A mRNA levels was conducted by TaqMan® probe real-time RT-PCR, as performed previously (Wang & Yang, 2016; Zhang et al., 2014). Briefly, RNA was isolated using a RNAprep Pure Tissue Kit (Tiangen Biotech, China) per the manufacturer's protocols. Real-time PCR mixtures were established with a One Step PrimeScript® RT-PCR Kit (Takara, Japan) and primers and probes for pIgR and IL-17A (Wang & Yang, 2016; Zhang et al., 2014). PCR was performed on a 7500 Real-Time PCR System with 7500 System SDS software version 1.4 (ABI, USA). GAPDH mRNA levels in all samples were used as internal controls.

### Confocal microscopy

Tissue sections were cut with a cryostat LEICA CM 1850 (Leica Inc., Germany) to a thickness of 20 microns. After removing the OCT with PBS supplemented with 0.1% Triton X-100 and FSG, the slides were washed with PBST and blocked with 10% normal goat serum for 1 h before incubation in polyclonal antibody against human pIgR (rabbit anti-PIGR, 4 µg/mL, Abcam, USA) at 4 ℃ overnight. Sections were incubated in secondary antibody (Alexa Fluor 488 conjugated goat anti-rabbit IgG, 2 µg/mL, Invitrogen, USA) for 1 h after washing off the extra primary antibody. Slides were washed and mounted with anti-fade mounting medium and observed with an Olympus FV1000D-ST confocal microscope (Olympus, Japan). Images (1024×1024) were acquired and morphometric measurements were obtained with Image-Pro Plus software version 6.0 (Media Cybernetics, Silver Springs, MD, USA).

### Statistics

All quantitative parameters were expressed as mean ± *SD*. Non-parametric Mann-Whitney *U* test was used to compare the means of parameters between normal and infected rhesus macaques. Spearman test was used to calculate the correlations between pIgR mRNA and IL-17A mRNA levels. *P* values of less than 0.05 were considered statistically significant.

## RESULTS

### Localization of pIgR immunoreactivity in the tracheal mucosa of rhesus macaques

**Figure 1:**
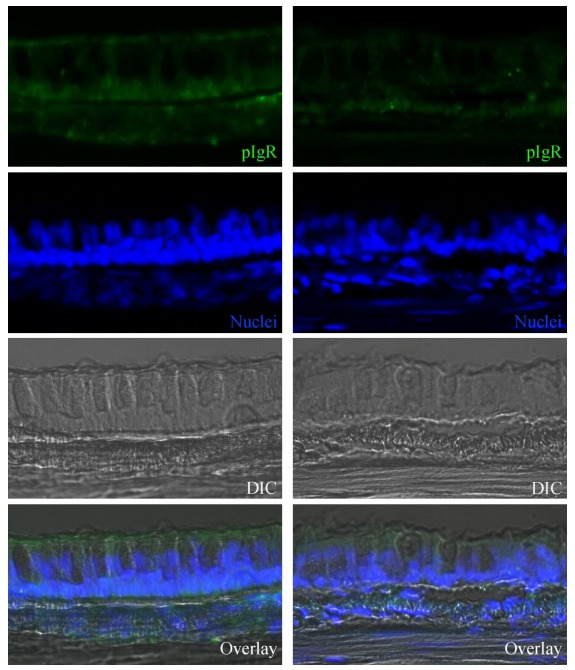
Distribution of pIgR immunoreactivity in the epithelium of tracheal mucosa from rhesus macaques

To detect the expression of pIgR in the tracheal mucosa of rhesus macaques, pIgR immunoreactive cells were examined with confocal microscopy. As shown in Figure 1, pIgR immunoreactivity was detected with a polyclonal antibody against human pIgR. In the epithelium, immunoreactivity to pIgR was localized to both the apical and basolateral surfaces of the epithelial cells. It was also localized in the cytoplasm of the basal part (under the nucleus) of the epithelial cells. After SHIV/SIV infection, pIgR immunoreactivity was lower in the tracheal mucosa of rhesus macaques.

### Expression of pIgR decreased in the tracheal epithelium of SHIV/SIV-infected rhesus macaques

**Figure 2:**
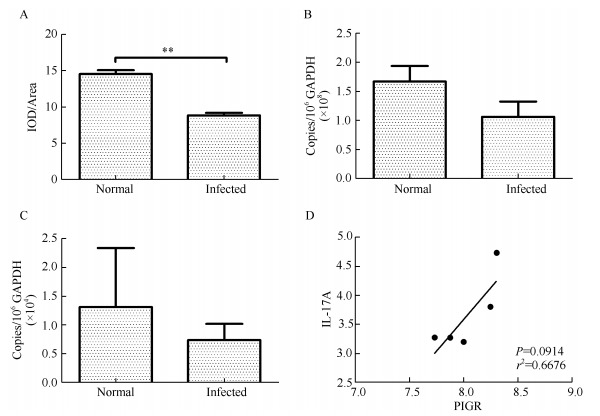
Expression levels of pIgR and IL-17A in the tracheal mucosa of rhesus macaques

To determine changes in pIgR expression after SHIV/SIV infection, levels of pIgR immunoreactivity were quantitatively examined with Image-Pro Plus software and pIgR mRNA levels were examined by real-time PCR. As shown in Figure 2, levels of pIgR immunoreactivity were 1.65 times higher in the tracheal epithelium of normal rhesus macaques than that in SHIV/SIV-infected rhesus macaques (Figure 2A), with statistical significance (Mann-Whitney *U* test, *P*=0.007 9). The transcription levels of pIgR genes in the tracheal mucosa of normal rhesus macaques were 1.57 times higher than that in infected rhesus macaques, although the difference was not statistically significant (Mann-Whitney *U* test, *P*=0.254 4). Therefore, both the transcription and protein levels of pIgR were about 1.6 times higher in normal than in infected rhesus macaques.

IL-17A is a regulator of pIgR expression and is decreased in HIV and SIV infection. We examined the transcription levels of IL-17A in the tracheal mucosa of normal and infected rhesus macaques. IL-17A mRNA levels in the tracheal mucosa of normal rhesus macaques were 1.8 times higher than that in SHIV/SIV-infected rhesus macaques (Figure 2C), although the difference did not reach statistical significance (Mann-Whitney *U* test, *P*=0.5476). Positive correlation was observed between pIgR and IL-17A mRNA levels in the tracheal mucosa of normal rhesus macaques (Figure 2D), though this trend was not found in SHIV/SIV-infected rhesus macaques.

### Expression of pIgR in the lungs of SHIV/SIV-infected rhesus macaques

**Figure 3:**
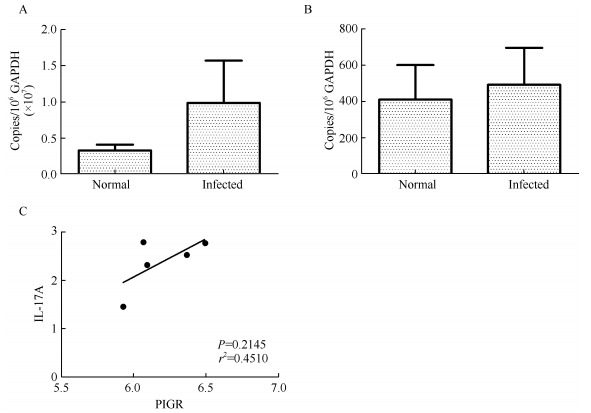
Expression of pIgR and IL-17A in the lungs of rhesus macaques

To determine whether the lungs of SHIV/SIV-infected rhesus macaques were similarly affected, the expressions of pIgR mRNA and IL-17A mRNA in the lungs of normal and infected rhesus macaques were examined. The mRNA levels of pIgR and IL-17A were 50 and 32 times higher, respectively, in the tracheal mucosa than in the lungs. As shown in Figure 3, pIgR and IL-17A mRNA were both detected in the lungs of normal and infected rhesus macaques. In contrast to the changes observed in the tracheal mucosa, the levels of pIgR and IL-17A mRNA were 3 and 1.2 times higher, respectively, in infected rhesus macaques than in normal rhesus macaques, although the differences were not statistically significant (Mann-Whitney *U* test, *P*=0.4396 and 0.7857, respectively). Therefore, the expressions of pIgR and IL-17A were higher in the tracheal mucosa than in the lungs, and were not reduced in the lungs of SHIV/SIV-infected rhesus macaques.

## DISCUSSION

In the present study, we observed reduced expression of pIgR in the tracheal mucosa of SHIV/SIV-infected rhesus macaques. Both the protein levels and mRNA levels of pIgR were decreased to almost the same degree, although the decrease in protein levels was statistically significant, whereas that of mRNA was not. It is possible that the effects of SHIV/SIV infection on pIgR expression were at the gene transcription level. In consistent with these results, previous research showed that pIgR mRNA levels were significantly reduced in the intestinal mucosa of SHIV/SIV-infected rhesus macaques (Wang & Yang, 2016). Downregulation of pIgR in airway mucosa has also been documented in other airway diseases (Gohy et al., 2014; Hupin et al., 2013). Since the pathology between SIV and HIV infection is similar, pIgR expression in the airway mucosa of HIV-infected patients could also be significantly affected.

The mechanism of decreased pIgR expression in SHIV/SIV infection has not been addressed. There are many potential factors that could affect pIgR expressiopn, among which IL-17A can significantly regulate pIgR expression (Jaffar et al., 2009). In the present study, a decrease in IL-17A expression in the tracheal mucosa of infected rhesus macaques was observed, suggesting a role of IL-17A in the downregulation of pIgR expression in the context of SHIV/SIV infection. The non-significant difference might be due to the large individual variability and small sample size. Significant correlation between pIgR and IL-17A mRNA has been observed in the intestinal mucosa of these animals and a significant decrease in IL-17A mRNA has also been observed in the intestinal mucosa (Wang & Yang, 2016; Zhang et al., 2014). Further studies are warranted to reveal the mechanism underlying the decrease of pIgR expression in HIV/AIDS.

The consequence of reduced pIgR expression in the tracheal mucosa of SHIV/SIV-infected rhesus macaques is unknown. Nevertheless, these data indicate impaired immune exclusion of potential pathogenic and commensal microbes in the respiratory system. In line with this, increased airway microbes and pulmonary infections have been documented in HIV/SIV infections (Nimmo et al., 2015; Twigg et al., 2016). Since elevated microbes can drive the COPD-like phenotype in pIgR deficient mice (Richmond et al., 2016) and downregulation of pIgR is observed in COPD patients (Gohy et al., 2014), reduced pIgR expression could be an underlying mechanism of the increased incidence of COPD in HIV/AIDS patients (Morris et al., 2011). COPD is the cause of death in a significant proportion of the HIV/AIDS population. ART treatment does not decrease the incidence of COPD, but is an independent predictor of increased airway obstruction (Gingo et al., 2010). Decreased expression of pIgR might also be involved in other pathological processes of HIV/AIDS, such as lung cancer (Ocak et al., 2012). Therefore, abnormal expression of pIgR should be taken into consideration in novel therapies for pulmonary complications such as COPD.

In summary, for the first time, reduced pIgR expression was observed in the tracheal mucosa of SHIV/SIV-infected rhesus macaques, which might be linked to IL-17A reduction in the tracheal mucosa. The reduced expression of pIgR might be the underlying mechanism of increased pulmonary microbiota and infections in HIV/AIDS. Rhesus macaques are a suitable model for future dissection of the mechanisms underlying respiratory complications in HIV/AIDS.

## References

[1] Gingo MR, George MP, Kessinger CJ, Lucht L, Rissler B, Weinman R, Slivka WA, Mcmahon DK, Wenzel SE, Sciurba FC, Morris A. 2010 Pulmonary function abnormalities in HIV-infected patients during the current antiretroviral therapy era. *American Journal of Respiratory and Critical Care Medicine*, 182 (6): 790- 796. 20522793 10.1164/rccm.200912-1858OC PMC2949404

[2] Gohy ST, Detry BR, Lecocq M, Bouzin C, Weynand BA, Amatngalim GD, Sibille YM, Pilette C. 2014 Polymeric immunoglobulin receptor down-regulation in chronic obstructive pulmonary disease.Persistence in the cultured epithelium and role of transforming growth factor-β. *American Journal of Respiratory and Critical Care Medicine*, 190 (5): 509- 521. 25078120 10.1164/rccm.201311-1971OC

[3] Grubb JR, Moorman AC, Baker RK, Masur H. 2006 The changing spectrum of pulmonary disease in patients with HIV infection on antiretroviral therapy. *AIDS*, 20 (8): 1095- 1107. 16691060 10.1097/01.aids.0000226949.64600.f9

[4] Hupin C, Rombaux P, Bowen H, Gould H, Lecocq M, Pilette C. 2013 Downregulation of polymeric immunoglobulin receptor and secretory IgA antibodies in eosinophilic upper airway diseases. *Allergy*, 68 (12): 1589- 1597. 24117840 10.1111/all.12274

[5] Jaffar Z, Ferrini ME, Herritt LA, Roberts K. 2009 Cutting edge:lung mucosal Th17-mediated responses induce polymeric Ig receptor expression by the airway epithelium and elevate secretory IgA levels. *The Journal of Immunology*, 182 (8): 4507- 4511. 19342622 10.4049/jimmunol.0900237 PMC2740792

[6] Johansen FE, Kaetzel CS. 2011 Regulation of the polymeric immunoglobulin receptor and IgA transport:new advances in environmental factors that stimulate pIgR expression and its role in mucosal immunity. *Mucosal Immunology*, 4 (6): 598- 602. 21956244 10.1038/mi.2011.37 PMC3196803

[7] Johansen FE, Pekna M, Norderhaug IN, Haneberg B, Hietala MA, Krajci P, Betsholtz C, Brandtzaeg P. 1999 Absence of epithelial immunoglobulin A transport,with increased mucosal leakiness,in polymeric immunoglobulin receptor/secretory component-deficient mice. *The Journal of Experimental Medicine*, 190 (7): 915- 922. 10510081 10.1084/jem.190.7.915 PMC2195652

[8] Morris A, George MP, Crothers K, Huang L, Lucht L, Kessinger C, Kleerup EC. 2011 HIV and chronic obstructive pulmonary disease:is it worse and why?. *Proceedings of the American Thoracic Society*, 8 (3): 320- 325. 21653535 10.1513/pats.201006-045WR PMC3132792

[9] Murray JF. 1996 Pulmonary complications of HIV infection. *Annual Review of Medicine*, 47 117- 126. 10.1146/annurev.med.47.1.117 8712766

[10] Nimmo C, Capocci S, Honeyborne I, Brown J, Sewell J, Thurston S, Johnson M, Mchugh TD, Lipman M. 2015 Airway bacteria and respiratory symptoms are common in ambulatory HIV-positive UK adults. *European Respiratory Journal*, 46 (4): 1208- 1211. 26113673 10.1183/13993003.00361-2015

[11] Ocak S, Pedchenko TV, Chen H, Harris FT, Qian J, Polosukhin V, Pilette C, Sibille Y, Gonzalez AL, Massion PP. 2012 Loss of polymeric immunoglobulin receptor expression is associated with lung tumourigenesis. *European Respiratory Journal*, 39 (5): 1171- 1180. 21965228 10.1183/09031936.00184410 PMC3717253

[12] Richmond BW, Brucker RM, Han W, Du RH, Zhang YQ, Cheng DS, Gleaves L, Abdolrasulnia R, Polosukhina D, Clark PE, Bordenstein SR, Blackwell TS, Polosukhin VV. 2016 Airway bacteria drive a progressive COPD-like phenotype in mice with polymeric immunoglobulin receptor deficiency. *Nature Communications*, 7 11240- 10.1038/ncomms11240 PMC4822073 27046438

[13] Twigg HL 3rd, Knox KS, Zhou J, Crothers KA, Nelson DE, Toh E, Day RB, Lin HY, Gao X, Dong QF, Mi DM, Katz BP, Sodergren E, Weinstock GM. 2016 Effect of advanced HIV infection on the respiratory microbiome. *American Journal of Respiratory and Critical Care Medicine*, 194 (2): 226- 235. 26835554 10.1164/rccm.201509-1875OC PMC5003215

[14] Wang Y, Yang GB. 2016 Alteration of polymeric immunoglobulin receptor and neonatal fc receptor expression in the gut mucosa of immunodeficiency virus-infected rhesus macaques. *Scandinavian Journal of Immunology*, 83 (4): 235- 243. 26860548 10.1111/sji.12416

[15] Zar HJ. 2008 Chronic lung disease in human immunodeficiency virus (HIV) infected children. *Pediatric Pulmonology*, 43 (1): 1- 10. 18041077 10.1002/ppul.20676

[16] Zhang WJ, Wang Y, Yu K, Duan JZ, Yao WR, Wang Y, Yang RG, Yang GB. 2014 Associated changes in the transcription levels of IL-17A and tight junction-associated genes in the duodenal mucosa of rhesus macaques repeatedly exposed to simian/human immunodeficiency virus. *Experimental and Molecular Pathology*, 97 (2): 225- 233. 25034324 10.1016/j.yexmp.2014.07.007

